# Establishment and Characterization of PCL12, a Novel CD5^+^ Chronic Lymphocytic Leukaemia Cell Line

**DOI:** 10.1371/journal.pone.0130195

**Published:** 2015-06-25

**Authors:** Andreas Agathangelidis, Lydia Scarfò, Federica Barbaglio, Benedetta Apollonio, Maria Teresa Sabrina Bertilaccio, Pamela Ranghetti, Maurilio Ponzoni, Gabriella Leone, Valeria De Pascali, Lorenza Pecciarini, Paolo Ghia, Federico Caligaris-Cappio, Cristina Scielzo

**Affiliations:** 1 IRCCS San Raffaele Scientific Institute, Division of Experimental Oncology, Unit of Lymphoid Malignancies, Milano, Italy; 2 Università Vita-Salute San Raffaele, Milano, Italy; 3 IRCCS San Raffaele Scientific Institute, Division of Experimental Oncology, Unit of B Cell Neoplasia, Milano, Italy; 4 IRCCS San Raffaele Scientific Institute, Lymphoma Unit, Department of Onco-Hematology, Milan, Italy; 5 IRCCS San Raffaele Scientific Institute, Pathology Unit, Milan, Italy; Cornell University, UNITED STATES

## Abstract

Immortalized cell lines representative of chronic lymphocytic leukemia (CLL) can assist in understanding disease pathogenesis and testing new therapeutic agents. At present, very few representative cell lines are available. We here describe the characterization of a new cell line (PCL12) that grew spontaneously from the peripheral blood (PB) of a CLL patient with progressive disease and EBV infection. The CLL cell origin of PCL12 was confirmed after the alignment of its IGH sequence against the “original” clonotypic sequence. The IGH gene rearrangement was truly unmutated and no CLL-related cytogenetic or genetic lesions were detected. PCL12 cells express CD19, CD20, CD5, CD23, low levels of IgM and IgD and the poor-outcome-associated prognostic markers CD38, ZAP70 and TCL1. In accordance with its aggressive phenotype the cell line is inactive in terms of LYN and HS1 phosphorylation. BcR signalling pathway is constitutively active and anergic in terms of p-ERK and Calcium flux response to α-IgM stimulation. PCL12 cells strongly migrate *in vitro* in response to SDF-1 and form clusters. Finally, they grow rapidly and localize in all lymphoid organs when xenotrasplanted in Rag2^-/-^γc^-/-^ mice. PCL12 represents a suitable preclinical model for testing pharmacological agents.

## Introduction

Chronic lymphocytic leukemia (CLL) is characterized by the clonal expansion and accumulation of mature monoclonal CD5^+^ B cells in the peripheral blood (PB), bone marrow (BM) and secondary lymphoid organs [1]. The development and progression of CLL are determined by causal and influential genes and by a dynamic cooperation between tumor cells and normal bystander cells within specific tissue microenvironments [2].

Although CLL primary cells are easily available in high numbers from the patient’s PB, they survive poorly *in vitro* and do not easily grow *in vivo* in animal models [3]. Moreover, they are difficult to transfect (e.g. with electroporation or Liposomes methods), thus limiting studies at both gene and protein levels [4]. These features underline the impact that CLL cell lines could have to the application of long-term functional studies and the testing of new therapeutic agents [3],[5].

Nevertheless very few *bona fide* CLL cell lines have been reported (rewieved in ref Rosen et al [6]) in contrast to other haematological tumors. This cell line scarcity may likely be ascribed to the resistance of CLL primary cells to Epstein-Barr virus (EBV) transformation [7],[8] both *in vivo* and *in vitro*. Though EBV infection is very frequent in humans, the risk entailed by EBV-induced B-cell proliferation *in vivo* is tightly controlled by the immune system (reviewed by Klein et al [9]). In rare occasions EBV can infect CLL cells, which in turn can be transformed in cell lines [6],[10]. Recently, *Rasul et al* observed that the acquisition of EBV by CLL cells *in vivo* reflects the clinical course of the disease at the time of infection [11].

An exhaustive genomic and phenotypic analysis of a panel of existing CLL cell lines and normal B-cell lymphoblastoid cells, claimed to be derived from the same donors (CLL-LCLs) [12], revealed that among 17 CLL cell lines analysed only 10 were of authentic neoplastic origin.

Here we describe the establishment and characterization of a new CLL cell line (PCL12) obtained from the PB of a CLL patient who had an on-going EBV infection. Thanks to its resemblance to CLL primary cells and its ability to grow *in vivo*, this novel cell line may become a useful tool to dissect CLL biology and immunogenetics and to screen new drugs.

## Materials and Methods

### Human Ethics Statement

CLL patient was diagnosed according to the updated National Cancer Institute Working Group (NCIWG) guidelines [13]. Peripheral blood samples were obtained after patient informed consent (written), as approved by the institutional ethics committee of San Raffaele University Hospital (Milano, Italy).

The study has been specifically approved by the OSR ethics committee in the protocol VIVI-CLL titled:”*In vivo* and *in vitro* characterization on CLL”

### Cell culture

Leukemic CD19 cells were negatively selected after blood withdrawal using the Rosette Sep B-lymphocyte enrichment kit (Stem Cell Technologies). Purity of the preparation was more than 99% and cells co-expressed CD19 and CD5 on their cell surfaces, as shown by flow cytometry (FC500; Beckman Coulter); the preparation was virtually devoid of natural killer (NK) cells, T lymphocytes, and monocytes [14].

Cells were cultured in RPMI 1640 medium (Invitrogen) supplemented with 10% volume/volume (v/v) Fetal Bovine Serum (FBS) and 15 mg/ml Gentamicin (complete RPMI) at 37°C, 5% CO_2_. The morphology of neoplastic population was evaluated on cytocentrifuged cells stained with Haematoxylin and Eosin.

### Flow cytometry

1x10^6^ PCL12 cells were stained for the following CD antigens: CD5, CD10, CD19, CD20, CD23, CD27, CD38, CD45, CD54, CD80, CD83, CD95, CD200, IgD, IgM, CXCR4, CXCR5, VLA4, HLA-DR, FMC7, ZAP70, TCL1 (BD Biosciences Pharmingen). For intracellular staining the FIX&PERM Kit (Beckman Coulter) was used and Kit instructions were followed. Expression levels were analysed using Cytomics FC500 (Beckman Coulter).

### Phospho-Flow Cytometry

ERK1/2 phosphorylation status was analyzed by flow cytometry on PCL12 cells. Briefly, single cell suspensions were fixed in Lyse/Fix Solution (BD Biosciences Pharmingen) for 10 minutes at 37°C. Cells were then washed and permeabilized by using a Perm Buffer (0.5% saponin, 5% FCS, 10mM Hepes) for 20 minutes at room temperature. Cells were washed and incubated with anti human CD19 and anti human pERK1/2 for 30 minutes at 4°C and analyzed with a FC500 flow cytometer. PMA-stimulated cells (10 minutes at 37°C) were used as positive control (data not shown).

### Antibodies used for Western Blot

Mouse α-HS1, mouse α-LYN and mouse α-SYK antibodies were purchased from BD Biosciences; mouse α-phosphoERK (Y204), rabbit α-ERK2 from Santa Cruz Biotechnology; mAb rabbit α-phosphoHS1 (Y397)-clone D12C1, rabbit α-phosphoLYN (Y507), rabbit α-VAV1, rabbit α-phosphoVAV (Y174); mAb rabbit α-phosphoLYN (Y396)-clone EP503Y from Epitomics. α-pPLC-γ -Y1271 and α-pPLC-γ, x-phosphoBTK (Y223) and α-BTK from Cell signaling. Primary antibodies were used at 1:1000 dilution. Anti-mouse IgG HRP-linked and anti-rabbit IgG HRP-linked (1:5000 dilution) were purchased from GE Healthcare.

### Inhibition and Stimulation

U0126 and 11R-VIVIT peptides were purchased from Calbiochem. As positive control for phospho-flow staining, cells were stimulated with PMA (100 ng/ml, Sigma) for 5 minutes. *In vitro* BCR triggering was performed by using 20 μg/ml Goat F(ab)2 α-human IgM (Invitrogen) for 5 minutes. In *vitro* kinase inhibition was performed by using Ibrutinib at 1 μM concentration from Selleckchem at different time points 5’, 30’, 1 hour and 24 hours.

### Cell lysis and Western blot (WB) analysis

Cells were lysed with ice cold Lysis Buffer (NaCl 0.15M; 1% NP40; 1mM EDTA pH = 8; 50mM Tris-HCl pH = 7, pepstatin, leupeptin, PMSF, sodium orthovanadate and NAF). Whole protein extracts were resolved by sodium dodecyl sulfate-polyacrylamide gel electrophoresis (SDSPAGE) and proteins were electron-transferred from the gel onto nitrocellulose membranes and incubated with the indicated antibodies.

Immunoreactivity was revealed by incubation with secondary antibodies conjugated with Horseradish peroxidise (HRP).

### Intracellular calcium flux

Intracellular calcium flux was measured by using the fluorogenic probe Fluo3AM (Invitrogen), as previously described [15].

Briefly, cells (at concentration of 10^7^ cells/ml in complete RPMI) were incubated with 4μM of Fluo3-AM and 0.02% (vol/vol) of Pluronic F-127 (Sigma) for 30 minutes at 37°C. Cells were then washed and resuspended at a concentration of 5x10^6^ cells/mL in complete RPMI at room temperature. Cells (250 μL) were incubated at 37°C for 5 minutes prior to the acquisition of background fluorescence (i.e, of unstimulated cells) followed by addition of 20 g/ml goat F(ab)2 anti–human IgM or IgD (Southern Biotechnology) and data acquisition for an additional 5 minutes.

Data were acquired on a FC500 and analyzed by using the FlowJo software (Tree Star).

### Immunofluorescence staining and confocal microscopy

Sterile glass slides were placed in a 24-well plate and coated with 400μl poly-L-ornithine (Sigma Aldrich) overnight at 4°C. After one wash with PBS, 500000 PCL12 cells were disseminated on the slides. After 2-hour incubation, cells were washed and fixed with 4% v/v PAF (Sigma-Aldrich) for 20 minutes at room temperature. After blocking (Blocking buffer: 0,1% w/v BSA, 10% v/v FBS in PBS), cells were permeabilized in Blocking Buffer containing 0.3% v/v Triton-X100 (Sigma Aldrich). Cells were incubated overnight with primary antibody (**α**-HS1 from Enzo Life sciences,) and stained for 2 hours with secondary antibody (**α**-mouse AlexaFluor488). Slides were then mounted using ProLong Gold antifade reagent with DAPI (Invitrogen) as mounting media. Images were acquired using Laser Scanning Confocal Microscope (LEICA) with an inverted 40x oil objective. Plot profile analysis was performed using Imagej Software.

### Chemokine mediated in vitro migration

Half a million PCL12 cells were seeded to a transwell chamber with a 6.5 mm diameter and 5.0 m pore size (Cornig Incorporated); after 4 hours cells migrated to the lower part of the chamber and were counted with the use of the cytometer (FC500). Chemokine mediated migration was evaluated adding 100ng α-SDF-1 to the lower chamber and the migration index was calculated (n° of cells migrated after chemokine addiction: (n° of cells migrated without x100) / (total n° of cells).

### Polymerization assay

One million PCL12 cells were pre-warmed at 37°C in RPMI w/o serum. After a 10-minute incubation with anti-IgM the reaction was terminated with the addition of 4% paraformaldehyde and cells were permeabilized with saponine 0.2% on ice. The percentage of F-actin was measured by flow cytometry using the Phalloidin-AlexaFluor633 (1:1000). F-actin increase was calculated as the result of: mean fluorescence intensity after anti-IgM stimulation/mean fluorescence time zero.

### Gene sequence amplification and mutation analysis

PCR amplification of the IGHV-IGHD-IGHJ gene rearrangements for both the CLL clone of the patient and the PCL-12 cell line was performed on genomic DNA (gDNA), using appropriate forward and reverse primers. PCR amplicons were subjected to direct sequencing on both strands. Sequences were analyzed using the IMGT/V-QUEST tool (http://www.imgt.org).

PCR amplification and Sanger sequencing of exons 14–16 of the SF3B1 gene and exon 5 of the MYD88 gene were performed using standard protocols, as previously described [16]. Exon 34 of the NOTCH1 gene was scanned for the presence of the NOTCH1 c.7544_7545delCT mutation by amplification refractory mutation system (ARMS) using the standard published protocol [16]. TP53 gene mutations were investigated through Direct Sanger sequencing of complementary DNA. Sequencing analysis was performed on both forward and reverse reactions. Analysis of mutations was performed with the Vector NTI Express Software (Life technologies). Primers and reaction conditions can be found at www-p53.iarc.fr.

### Karyotyping and FiSH

Cell cultures were treated with colcemid (GibcoKaryoMAXColcemid solution in PBS, LifeTechnologies) at a final concentration of 10ng/mL for 16 hours (overnight) at 37°C and metaphases harvest was carried out according to standard protocols. Briefly, PBS washed cells were treated with hypotonic solution (0.075 M KCl for 15min at RT) and fixed in acetic acid/methanol (1:3 v/v). Air-dried metaphase spreads slides were analysed by QFQ banding following standard procedures; 25 metaphases were analyzed and description of karyotypes and clonality criteria followed the International System for Human Cytogenetic Nomenclature recommendations [17],[18].

Both patient whole peripheral blood and cell line cells underwent interphase FISH analysis after 10 months in culture, according to standard protocols. The FISH panel included probes for the detection of trisomy 12, deletions of 11q22.3 (ATM), 13q14.3 (D13S319), 13q34 (LSI13q34), and 17p13 (TP53) (Vysis LSI p53/LSI ATM and LSI D13S319/LSI 13q34/CEP 12 Multi-color Probe; Abbott Molecular) and 200 interphase nuclei were counted for each probe. Cutoffs were set at 2% for trisomy 12 and 5% for deletions. For both classic and molecular cytogenetics analysis microscope observation was performed using a Nikon Eclipse 90i (Nikon Instruments, Japan) equipped with the acquisition and analysis Genikon software (Nikon Instruments S.p.a. Italy).

### Animal Ethics Statement

All animal work and care were performed under the guidelines and approval of San Raffaele Scientific Institute (HSR) ethics committee (namely:” Il comitato istituzionale per la buona sperimentazione animale dell’ospedale San Raffaele) and in accordance with the recommendations of the Guide for the Care and Use of Laboratory Animals and with the approval of the Committee on the Ethics of Animal Experiments (IACUC) and finally approved by the Italian Ministry of Health Regione Lombardia. Specific authorization for the mouse experiments performed in this work (injection of Human CLL cells in Rag2^-/-^γ _c_^-/-^ to follow grow and dissemination paragraph 2.1) was obtained in the protocol #601 titled: “Study of the interaction between neoplastic B cells and the microenvironment in Lymph proliferative disorders by the use of humanized mouse models”. Authors Cristina Scielzo, Maria Teresa Sabrina Beritilaccio, Benedetta Apollonio and Paolo Ghia are elected responsible for the experiments.

All reasonable efforts were made to ameliorate animal suffering, including general anaesthesia for painful procedures performed by inhalation in a dedicated chamber of a mixture made by CO2+O2 (50%+50%) before injection of the cells. To sacrifice the mice CO2 inhalation was used by staff specifically trained accordingly with the protocol proposed for this study and approved by the ethics committee (IACUC) and the Italian Ministry of Health Regione Lombardia.

### *In vivo* experiments

Eight-week-old Rag2^-/-^γ _c_^-/-^ female mice were challenged subcutaneously in the left flank with 10×10^6^ PCL12 cells in 0.1 ml of saline through a 27-gauge needle, as previously described.

Animals were monitored twice a week for weight and tumor growth (measuring 3 perpendicular diameters) and were sacrificed when the mean tumor volume reached 1000 mm^3^ or when animals experienced clinical signs of illness following the criteria approved and described in the protocol #601 (see Animal Ethics Statement). Peripheral blood and/or tissue (spleen, lymph nodes and femoral bone marrow) single cell suspensions were depleted of red blood cells by incubation in an ammonium chloride solution (ACK) lysis buffer (NH4Cl 0.15 M, KHCO3 10 mM, Na2EDTA 0.1 mM, pH 7.2–7.4) and were then stained after blocking the fragment crystallizable (Fc) receptors. After blocking Fc receptors with Fc block (BD Biosciences Pharmingen) for 10 minutes at room temperature to avoid nonspecific binding of antibodies, cells from peripheral blood, bone marrow, peritoneal exudates, lymph nodes (when present) and spleen were stained with anti human CD19 antibody, to investigate the presence of PCL12 cells in the different compartments, and analyzed with a FC500 flow cytometer (Beckman-Coulter).

### Immunohistochemistry

Mice sections were de-paraffinized in xylene, rehydrated in ethanol, immersed in 0.1 M citric acid pH 6.0, heated in microwave and cooled at room temperature. Endogenous peroxidases were quenched with 3% H_2_O_2_. Slides were incubated with 3% BSA and with primary monoclonal antibodies: CD20, CD5, CD23, CD19. Slides were incubated with streptavidin 1:500 for 30 min. Slides were then incubated with 3,3’-diaminobenizidine tetrahydrochloride (DAB) for 5 min and the counterstain was done with Mayer-Hematoxylin. After dehydratation in ethanol and xylene, slides were permanently mounted in Eukitt (Bio-Optica).

### Statistical analysis

Student's paired or unpaired t-tests were used for statistical comparisons. Comparison of tumor grow was performed with the use of the log-rank test. Analyses were performed using GraphPad Prism 4 software (GraphPad Software, San Diego, CA, USA). A P-value <0.05 was considered as statistically significant.

## Results

### Establishment of the PCL12 cell line

A caucasian male 73 years old diagnosed with Rai stage I, Binet stage A CLL in 2004 received over the years multiple treatments including FCR (fludarabine, cyclophosphamide and rituximab), BR (bendamustine+rituximab), CHOP (cyclophosphamide, doxorubicin, vincristine and prednisone) followed by rituximab maintenance until January 2012, when he was referred to our Center. He remained untreated until September 2012, when, admitted to the hospital because of fever of unknown origin and a progressive disease characterized by increased lymphadenopathies, was found to have an EBV infection and died one month later because of septic shock.

**Fig 1:**
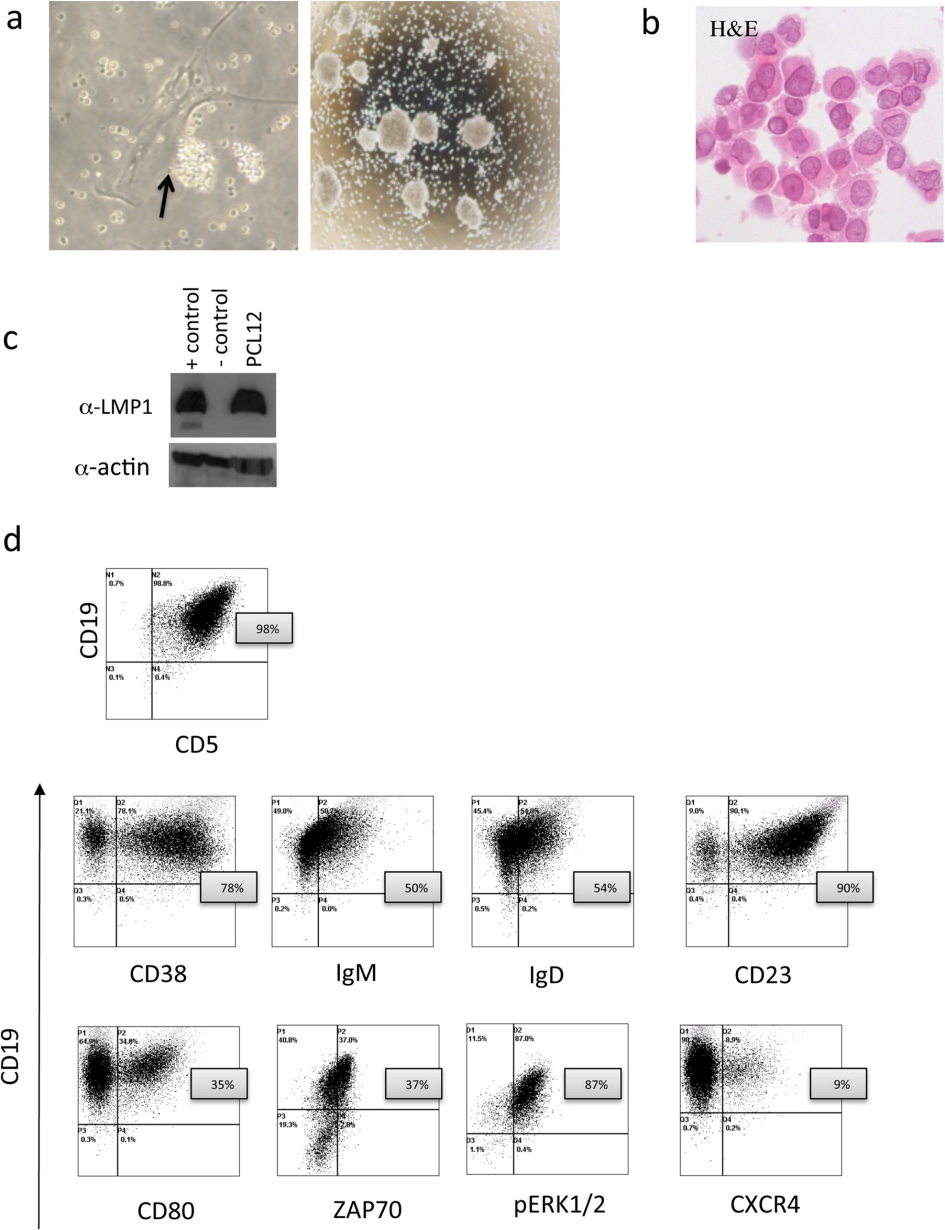
(a) PCL12 cells images acquired with Nikon TS100 microscope after 4 weeks of culture (20x magnification objective in the left panel), dark arrow indicating nurse like cells, and 6 weeks of culture (4x magnification objective on the right). (b) Haematoxylin and Eosin staining (c) WB analysis for LMP1 protein on PCL12 cells, Positive control JY cell line and negative RL cell line. (d) PCL12 immunophenotype dot plot acquired by Flow cytometry.

Peripheral blood mononuclear cells (PBMC) were obtained by gradient separation after admission. We seeded 3x10^6^ total PBMC and 3x10^6^ purified CD19^+^ cells in RPM1 medium supplemented with 10% FBS and 15 mg/ml Gentamicin in 25 cm^2^ filtered flasks and followed cell growth over time. While purified CD19^+^ cells died after 2 weeks of culture, in the total PBMC cultures we observed, after 4 weeks the persistence of mononuclear cells that tended to form clusters in close contact with adherent cells, the latter being likely nurse-like cells [19], (Fig 1A left panel). After 2 additional weeks, cells started to grow in suspension forming clumps. These cells were CD19^+^CD5^+^ (98%) (Fig 1D) and they rapidly expanded when transferred to a new flask (Fig 1A right panel). Cells have been maintained in culture for over 1 year without any difficulty. The cell morphology was evaluated with Haematoxylin and Eosin staining (Fig 1B). We tested the cell line for the presence of EBV viral proteins and we observed high LMPI protein expression (Fig 1C).

The newly generated cell line has been named PCL12.

### PCL12 cell line immune-phenotype

We analysed a number of surface and intracellular markers (Fig 1D and Table 1) and we observed the expression of the classical B cell markers CD19 and CD20 on 100% of the cells. CD5 was present in 98% cells, together with CD23 (90%), low levels of IgM (50%, MFI: 4,4) and IgD (54%, MFI: 20). Activation markers such as CD95 (95%), CD80 (bimodal 35%) and the memory B-cell marker CD27 were present (98%). PCL12 cells exhibited a bimodal expression of CD38 (78%) and were positive for ZAP70 (37%) and for CD200 expression (25,6%) [20].

**Table 1:** Detailed immunogenetic, genetic and cell phenotype information of the PCL12 cell line and comparison to the respective properties of the MEC1 and MEC2 cell lines.

	PCL12	MEC1	MEC2
**Immunogenetics**	V3-30-3 | D2-15 | J6	V4-59 | D2-21 | J4	
	100%	94.65%	
	AREGALAGDIVVVVAANYYYYYGMDV	ARSQGVLTAIDY	
	26	12	
	Igλ (V2-8 | J2/3)	Igκ (V4-1 | J2)	
	no mutations	c.7544_7545delCT	-
**Genetics**	no mutations	no mutations	-
	R72 polymorphism	R72 polymorphism	-
	c.521C>G, p.P174T	c.949_950insC	-
	no mutations	no mutations	-
	no	yes	-
**Cell markers**	95%	0%	0%
	0%	0%	0%
	100%	99%	99%
	92%	96%	96%
	98%	76%	67%
	90%	89%	36%
	98%	99%	
	78%	90%	99%
	97%	99%	92%
	35%	98%	91%
	40%	4%	
	90%	90%	63%
	26%	0%	
	54%	97%	99%
	50%	99%	94%
	43%	99%	97%
	0%	8%	72%
	37%	18%	
	9%	5%	
	42%	12%	
	90%	98%	
	72%	98%	98%
	87%	Positive WB ^28^	

We also evaluated the expression of molecules involved in cell trafficking, homing and adhesion such as CXCR4, CXCR5, VLA4, Integrin β7 and CD54, all expressed at variable levels (Table 1). Using phospho-flow, we observed that the ERK1/2 proteins were constitutively activated. All reported markers were stably expressed over the whole period of *in vitro* culture.

Interestingly and in line with the aggressive phenotype of the cell line we observed high levels of TLC1 protein (83%) expression by flow cytometry (S1 Fig) [21] [22].

### Karyotype and FISH analysis showed no clonal abnormalities

Cytogenetic analysis of PCL12 after 10 months in culture revealed a diploid modal number and normal karyotype (46, XY); only one metaphase was 92, XXYY and 18 metaphases had one or two non recurrent structural rearrangements (terminal deletions, fragments of chromosomes, chromatide breaks, terminal fusions). No clonal aberrations were observed (S2 Fig). FISH analysis for trisomy 12 and deletions of 11q22.3 (ATM), 13q14.3, 13q34 and 17p13 (TP53) showed no abnormalities. The same FISH results were obtained by analyzing the patient’s PB.

### IG sequence analysis demonstrates the tumor origin of the PCL12 cell line and an unmutated IGHV gene status

The IGH gene rearrangement sequence expressed by the PCL12 cell line was aligned against the clonotypic rearrangement expressed by the CLL patient with the use of the ClustalW2 and IMGT/V-QUEST tools, in order to confirm its origin. Indeed, both IGH rearrangements were identical in terms of nucleotide composition and, thus, consisted of the same IGH genes and carried an identical heavy complementarity determining region 3 (VH CDR3) (S3 Fig).

**Fig 2:**
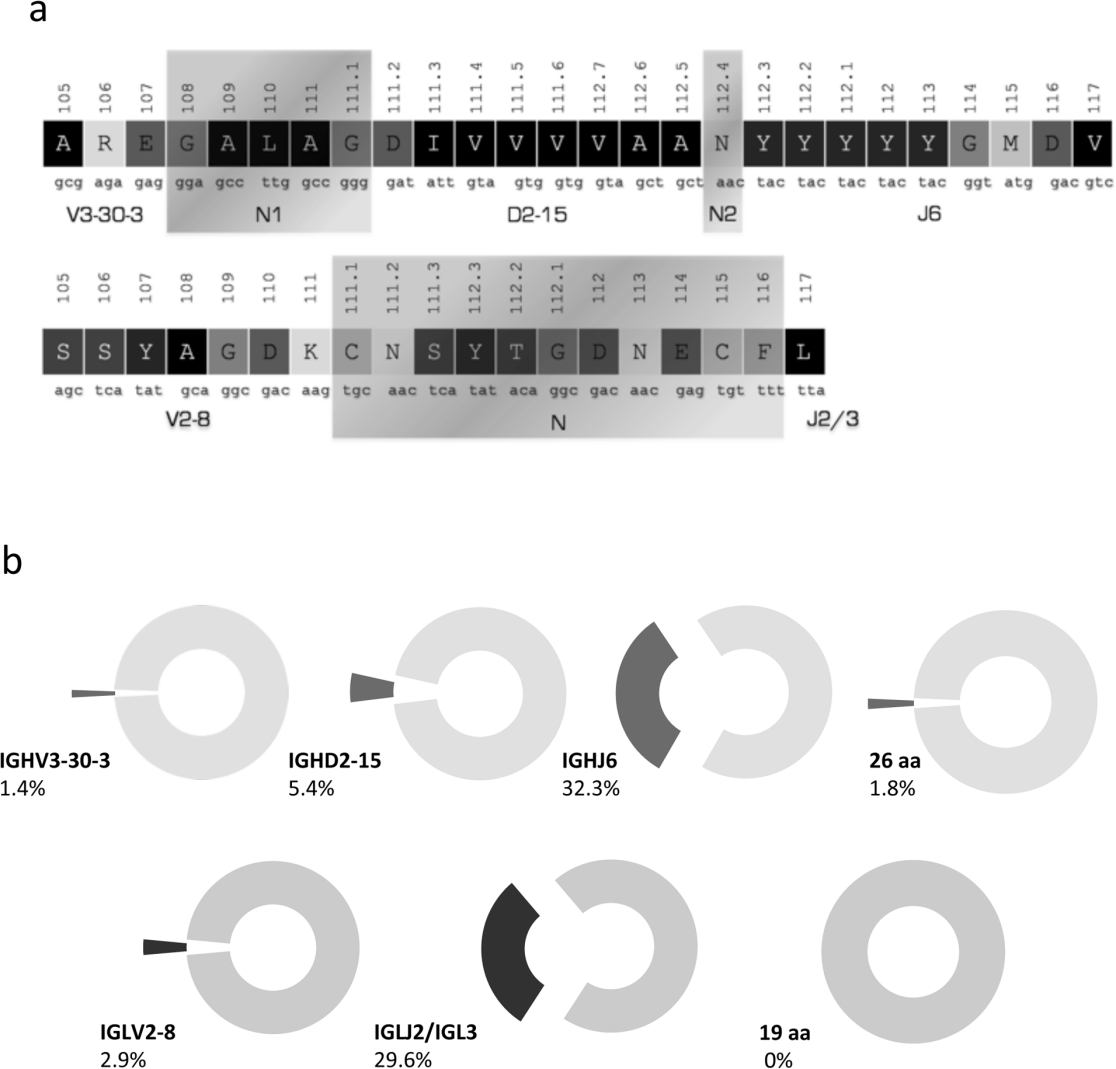
(a) VH and VL CDR3 amino acid composition and junction analysis. (b) Frequency analysis for the IG genes and the length of the CDR3 of the heavy and light chain of the BcR IG, respectively. Analysis performed on IG data from the largest up-to-date CLL cohorts [23].

In detail, both rearrangements were found to express the gene combination IGHV3-30-3/IGHD2-15/IGHJ6. Regarding the SHM status of the rearrangement no mutations were identified across the coding region of the rearranged IGHV gene. The VH CDR3 of the heavy chain was extremely long, comprising 26 amino acids (aa). Regarding the IG light chain, the IGLV2-8 gene was rearranged to the IGLJ2/3 gene, again producing a very long light CDR3 (VL CDR3) of 19 aa. In terms of SHM, the rearranged light chain was significantly mutated with a germline identity (GI) of 93.4% (Fig 2A).

Based on the data from the two largest studies on the heavy and light chain IG gene repertoires of CLL including 7596 IGH [23],[24] and 763 IGL gene rearrangements, respectively, the IGHV3-30-3 is rarely expressed by CLL cells (104/7596 sequences, 1.4%) and in most cases it does not bear somatic hypermutations (55/104 sequences, 53%). Regarding the whole gene rearrangement, the exact gene combination was not found among the 7596 rearrangement sequences of the cohort, even though the usage of both the IGHD2-15 and IGHJ6 genes is frequent in CLL (5.4% and 32.3%, respectively). A 26 aa long VH CDR3 is very rare in CLL and was found only in 1.8% of the cohort (136/7596 sequences), which was also the case when we focused our analysis on IGHV3-30-3 cases (4/104 sequences, 3.8%). The extreme length of the VH CDR3 prompted us to check with the IMGT/Junction Analysis tool for the possible presence of a D-D fusion, but the results were negative. The length of the VH CDR3 is in part due to the IGHJ6 usage, which is the longest IGHJ gene, but mainly to the presence of 6 aa in the N1 region. Within the set of 6759 CLL IGH sequences carrying a N1 region the median length of this region was 2 aa and a 6 aa N1 region was found only in 275 cases (4.1%) (Fig 2A).

The IGLV2-8 gene accounted for 2.9% (22/763 sequences) of the cohort and 8.3% (22/266 sequences) of the cases that expressed a lambda light chain. In the vast majority of cases it was found in the mutated form (GI<98%), in frequent combination with the IGLJ2/3 gene (16/22, 73%). The VL CDR3 of the PCL12 cell line (19 aa) was found as the longest after its comparison against the CLL cohort. None of the 266 lambda expressing CLL cases had a VL CDR3 longer than 14 aa. In this case, the great VL CDR3 length was mostly attributed to the N region that comprised 7 aa (Fig 2B).

### Gene mutation analysis showed a TP53 polymorphism

We scanned the PCL12 cell line for a series of gene mutations recently reported in CLL [25]. No somatic mutations were found within exons 14–16 of the *SF3B1* gene and exon 5 of the *MYD88* gene. Exon 34 of the *NOTCH1* gene was negative for the presence of the c.7544_7545 CT deletion. The *TP53* gene carried an arginine (R) at the polymorphic codon 72, whereas a fraction of cells carried a G to C mutation at position 521, which in turn led to an R to threonine (T) aa change at codon 174 that is a mutation hotspot, according to the UMD p53 database. Such mutation is reported in the IARC TP53 Database associated to a bone cancer, while it is not present neither in the dbSNP nor in a cohort of 1173 CLL patients recently analysed for TP53 mutations [26].

### BCR signalling pathway features

**Fig 3:**
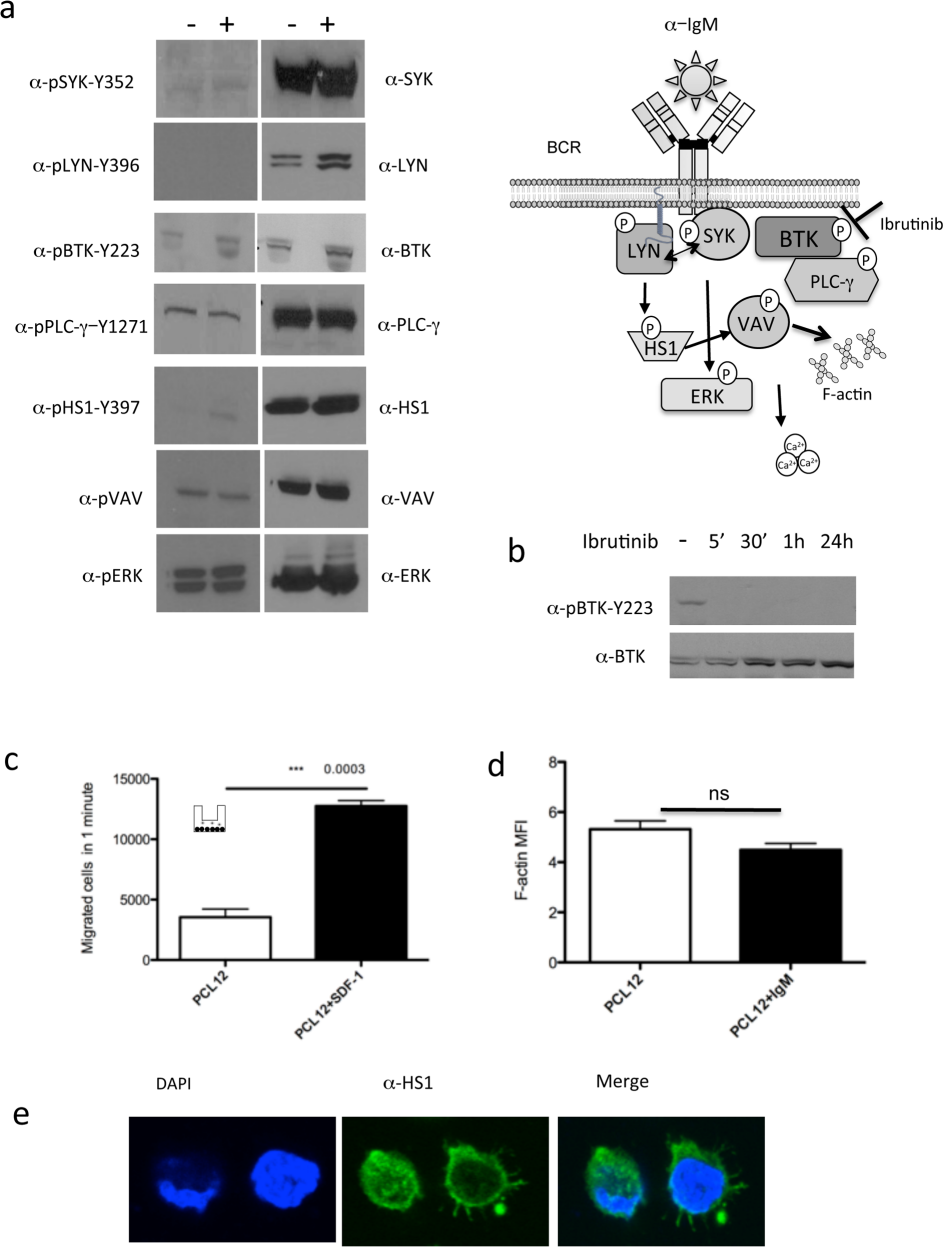
(a) WB analysis of PCL12 cells, basal (-) and after IgM stimulation (+), for BCR signaling pathway proteins activation and expression. On the right a schematic representation of the BCR signaling pathway analyzed. (b) WB analysis after treatment with ibrutinib (5’, 30’, 1 hour, 24 hour) for BTK activation.(c) Transwell PCL12 migration assay of migration with and without SDF-1 stimulation. (d F-actin polymerization index on PCL12 cells in basal conditions and after IgM stimulation (e) confocal analysis of PCL12 cells stained for HS1 protein in green and DAPI in blue.

We dissected the BCR signalling pathway of the PCL12 cells before and after IgM stimulation investigating a number of key molecules [27] (see schematic representation Fig 3A). We observed a constitutive activation of ERK1/2 by phospho-flow analysis (Fig 1D) (confirmed by western blot, Fig 3A) and stable intracellular levels of calcium (S4 Fig) after α-IgM stimulation. These findings indicate that PCL12 cells have a partially constitutively activated phenotype, a notion further supported by the constitutive presence of p-VAV, p-BTK and p-PLC-γ. On the contrary, SYK and LYN kinases and the HS1 protein were not active and could not be phosphorylated upon α-IgM stimulation, beside a very modest phosphorylation of HS1 Y397 (Fig 3A).

### PCL12 shows a dynamic cytoskeletal activity in terms of migration

PCL12 cells were found to have a very active migratory capacity *in vitro* in response to SDF-1, indicating their ability to respond to microenvironmental stimuli (Fig 3C). Accordingly, PCL12 cells showed a high basal level of F-actin polymerization activity that was not increased upon α-IgM stimulation (Fig 3D). Cells definitely adhered to the slides by forming long filopodia as highlighted by the expression of HS1 (Fig 3E).

### PCL12 as a tool to test novel therapeutic agents

Considering the constitutive activation of some BcR signalling mediators, we tested the activity of different inhibitors on PCL12 cells. Cells were treated with increasing doses of the MEK1/2 inhibitor U0126 and the NF-AT inhibitor VIVIT, which were both shown to revert anergy and induce apoptosis in a subset of CLL patients [28]. Treatment with both inhibitors reduced ERK1/2 phosphorylation after 2 hours of treatment and decreased cell viability after 48 hours. Specifically U0126 had an IC50 (half maximal inhibitory concentration) of 3 μM, while VIVIT had an IC50 of 9.5 μM (data not shown). We selected Ibrutinib, the most used BTK kinase inhibitor in CLL therapy [29], to test its effect on PCL12 cells and especially on BTK activation. We observed, already after 5 minutes of treatment, BTK de-phosphorylation which was also maintained after 24 hours of *in vitro* culture (Fig 3B),providing the proof of principle that PCL12 cells may represent a suitable preclinical model for testing pharmacological agents.

### PCL12 cells engraft *in vivo* in immuneodeficient mice

**Fig 4:**
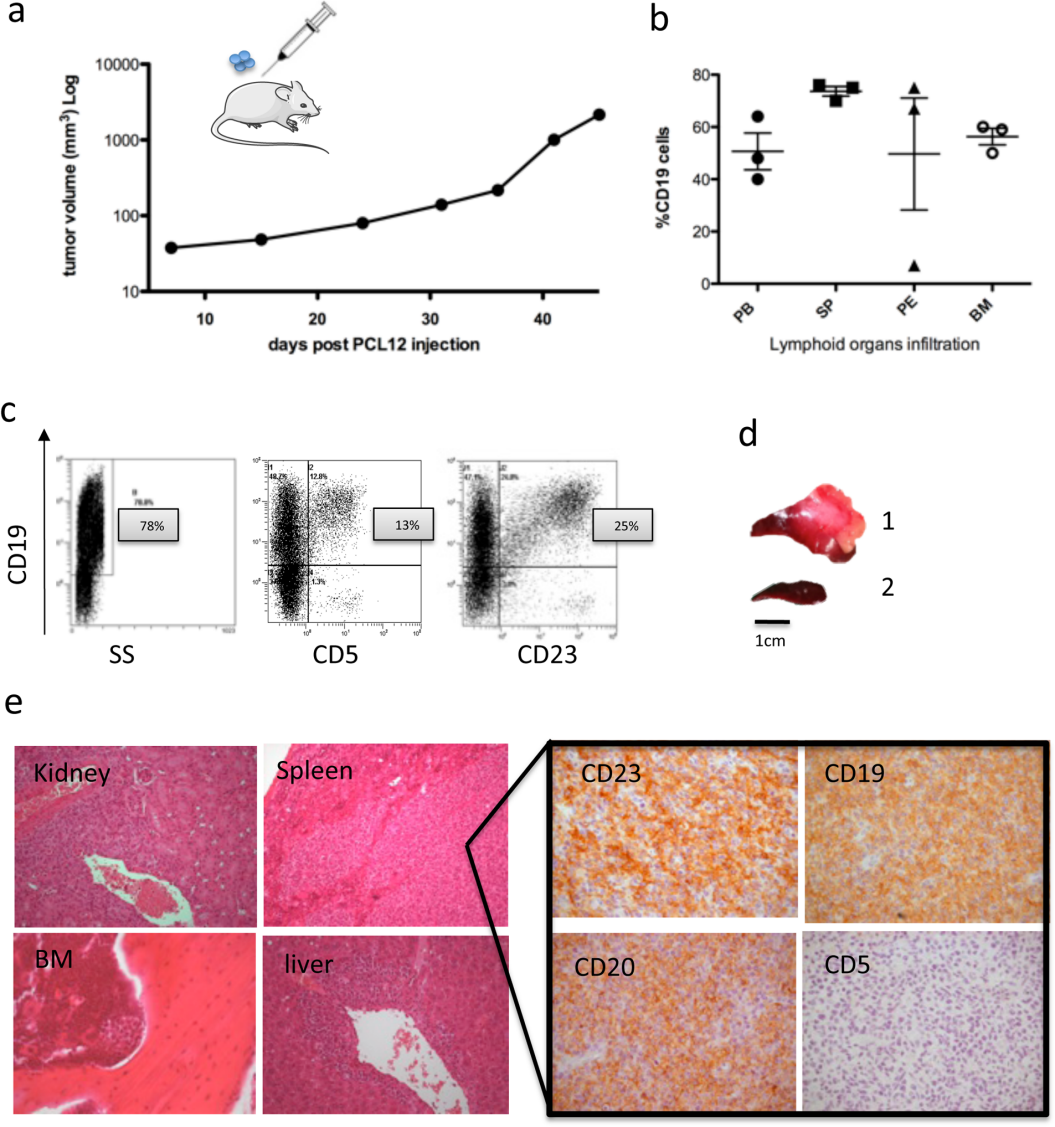
(a) Graph showing the subcutaneous tumor volume grow (b) CD19+ PCL12 cells infiltrating lymphoid organs quantified by flow cytometry in PB, SP, PE and BM. (c) Flow cytometric analysis of PCL12 cells in PB, surface CD19, CD5 and CD23 are represented. (d) images of the representative spleen from 1 mouse injected with PCL12 cells (1) and a WT (2). Histopathological examination of different tissues and IHC analysis.

We evaluated PCL12 cell capacity to grow in a Rag2-/-c-/- mouse model [5]. Mice were subcutaneously injected with 10x10^6^ PCL12 and the *in vivo* cell growth was monitored by measuring tumor volume over time (Fig 4A). Mice were sacrificed approximately 45 days after the injection when the tumor volume reached 1000 mm^3,^

At sacrifice, organs and lymphoid tissues (spleen, bone marrow, peritoneal exudate, and peripheral blood) were collected, processed and stained for human CD19, CD5 and CD23 markers, to confirm the presence of PCL12 cells.

PCL12 cells were able to engraft (Fig 4B) and maintained the expression of CD19 *in vivo*, while, interestingly, both CD5 and CD23 were down-regulated and a small proportion of cells retaining the expression of CD5 (13%) and CD23 (25%) were detectable only in the spleen, where PCL12 cells showed a preferential localization, but not in the other analysed organs. These findings were confirmed by immunohistochemistry (Fig 4E).

### Comparison of PCL12 against the well-established MEC1 & MEC2 cell lines

The PCL12 cell line properties were examined against those of the MEC cell lines. This comparison extended to 3 different levels, immunogenetic, genetic/genomic and cellular (Table 1). Concerning the properties of the BcR IG the cell lines are completely distinct: they use distinct IGH genes with the rearranged IGHV gene carrying a heavy mutational load in the MEC cell lines contrasting the absence of somatic mutations in the PCL12. Important differences were also identified within the VH CDR3 in terms of length and amino acid composition. Concerning the IG light chain PCL12 expresses a lambda-encoded light chain while the MEC cell lines express kappa.

The genetic background of the cell lines was comparatively examined for the presence of genetic lesions frequently reported in CLL. PCL12 cells carry a polymorphism (arginine at position 72, R72) and a mutation within the coding region of the *TP53* gene (c.521C>G, p.P174T). MEC1 cells exhibit a more complex genome comprising: (i) a 2 base-pair (bp) deletion within the *Notch1* gene and (ii) the same polymorphism (R72), a single bp insertion (c.949_950insC) and del17p13, all 3 affecting the *TP53* gene.

In terms of cell phenotype, PCL12 cells display a typical CLL cell phenotype: CD19+CD20+CD5+CD23+IgM^low^IgD^low^, in clear contrast to the MEC1 and MEC2 cells that express high levels of IgM and IgD and do not express CD5. Moreover, MEC2 cells also showed low CD23 expression. Other major differences concerned the activation markers CD80 and CD83 with the first being highly expressed in both MEC cell lines and the latter being more expressed in the PCL12 cell line. All cell lines were positive for CD38 with PCL12 being also positive for the expression of ZAP70. PCL12 cells also expressed higher levels of the cell trafficking marker CXCR5 compared to the MEC1 cells. In terms of BcR signaling both the PCL12 and the MEC1 cell lines showed activation of ERK.

## Discussion

In this paper we report the detailed characterization of PCL12, a novel CLL cell line that grew spontaneously in culture from the malignant B cells of a CLL patient carrying an EBV infection. The patient’s clinical infection, during disease progression, may have played a role in the immortalization of CLL clonal cells that are usually not prone to EBV infection. The origin of the PCL12 cell line was established by comparing its BcR IGH rearrangement sequence against the original clonal IGH sequence of the patient. After confirming the CLL-origin of the cell line, we analyzed in-depth the immunogenetic properties of the BcR IG compared with the other few existing CLL cell lines [12]. PCL12 BcR IG is different in almost any aspect: it expresses the IGHV3-30-3 gene with no somatic hypermutations (a truly unmutated status [GI = 100%][24]) and has a very long VH CDR3 (26 aa). Previously developed CLL express various IGHV genes usually in a mutated status (GI<98%) and carry VH CDR3 with a median of 16 aa. Only the WaC3CD5+ cell line exhibited similar immunogenetic properties: same IGHV and IGHJ genes, unmutated mutational status (no available information exists regarding the exact GI%) and a long VH CDR3 (25 aa). Yet, due to the usage of a different IGHD gene and the presence of different N-nucleotides the VH CDR3 sequence of the PCL12 was highly distinct. Furthermore, the BcR IG of PCL12 contains a lambda light chain in contrast to the kappa light chain of WaC3CD5+.

Another important feature of PCL12 cells is their normal karyotype while all the other CLL with a neoplastic origin reported carry cytogenetic aberrations. The observed non recurrent structural rearrangements are consistent with telomere dysfunction, perhaps due to EBV infection and consequent chromosomal instability [17].

It is relevant that PCL12 cell line maintains the expression of all the main surface markers *in vitro* and *in vivo* with the notable exception of CD5 and CD23, which is consistently present *in vitro*, while it is significantly down-modulated *in vivo*. The biological significance of this phenomenon, which indicates the need of *in vivo* interactions likely present only in the human microenvironment in contrast to the murine system, needs to be further explored.

PCL12 cells have an aggressive phenotype based on CD38, ZAP70 and TCL1 expression (molecular marker of poor clinical prognosis) a notion further corroborated by the presence of inactive LYN and HS1 as shown in patients with poor clinical out-come [30] as well as by the composition of the BcR IG. As documented by recent studies on BcR reactivity, unmutated BcR IG display a broader reactivity profile than mutated BcR IG, which in turn could account for an aggressive clinical disease course in patients with CLL. The role of Ag interactions in the PCL12 cell survival and expansion is revealed by the expression of hallmarks of ongoing activation such as CD80 and CD95.

At the functional level, PCL12 cells remain able to respond to external stimuli, such as SDF-1, though they are essentially anergic in terms of response to IgM stimulation, showing constitutive ERK activation. The latter findings have been also reported in a subgroup of patients with CLL who have an indolent clinical presentation and could be the result of the aforementioned ongoing activation through Ag interactions.

PCL12 cells appear to be a suitable preclinical model for testing pharmacological agents, as demonstrated by the *in vitro* response to the MEK1/2 inhibitor U0126 and the NF-AT inhibitor VIVIT, which were both shown to revert anergy and induce apoptosis in a subset of CLL patients [28]. Moreover PCL12 cells respond to treatment with Ibrutininb by stably inactivating BTK activity. This data indicates that PCL12 might be used as an additional tool to investigate the efficacy and the mechanism of action of different compounds. Moreover, the expression of surface markers such as CD20 and CD19 together with the ability to grow *in vivo* in a xenograft model point out to the possibility of using this cell line to study monoclonal antibody therapies alone or in combination with BCR signalling kinase inhibitors *in vivo*.

Finally, an extensive comparison was performed between PCL12 and the MEC1 and MEC2 cell lines in order to assess their relatedness, if any. Based on our findings, PCL12 is highly diverse under many of the aspects studied (immunogenertics, genetics, cell phenotype), indicating that, indeed, it represents a novel, distinct model to study CLL biology.

The PCL12 cell line will be made available to all investigators upon request.

## Supporting Information

S1 Fig Histogram shows TCL1 expression by Flow cytometry. (TIF)

S2 Fig (a) Representative metaphase, (b) showing normal karyotype 46, XY (c) a representative FISH result showing a normal signal pattern of ATM and p53 loci (ATM loci labeled with Spectrum Green and p53 loci labeled with Spectrum Orange); (d) a representative FISH result showing normal signal pattern for 13q14.3, 13q34 and CEP12 (labeled with Spectrums Orange, Green and Aqua, respectively). (TIF)

S3 Fig PCL12 IG sequence analysis to demonstrate the tumor origin of the cell line. (TIF)

S4 Fig Calcium Flux analysis. (TIF)
