# Correction: Establishment and Characterization of PCL12, a Novel CD5+ Chronic Lymphocytic Leukaemia Cell Line

**DOI:** 10.1371/journal.pone.0134748

**Published:** 2015-07-29

**Authors:** Andreas Agathangelidis, Lydia Scarfò, Federica Barbaglio, Benedetta Apollonio, Maria Teresa Sabrina Bertilaccio, Pamela Ranghetti, Maurilio Ponzoni, Gabriella Leone, Valeria De Pascali, Lorenza Pecciarini, Paolo Ghia, Federico Caligaris-Cappio, Cristina Scielzo

**Table 1:** “Detailed immunogenetic, genetic and cell phenotype information of the PCL12 cell line and comparison to the respective properties of the MEC1 and MEC2 cell lines,” is missing a column. Please see the corrected Table 1 here.

		PCL12	MEC1	MEC2
**Immunogenetics**	**IGH rearrangement**	V3-30-3 | D2-15 | J6	V4-59 | D2-21 | J4	
	**IGHV mutation status**	100%	94.65%	
	**VH CDR3**	AREGALAGDIVVVVAANYYYYYGMDV	ARSQGVLTAIDY	
	**VH CDR3 length**	26	12	
	**IGL rearrangement**	Igλ (V2-8 | J2/3)	Igκ (V4-1 | J2)	
**Genetics**	**NOTCH1**	no mutations	c.7544_7545delCT	-
	**SF3B1**	no mutations	no mutations	-
	**TP53**	R72 polymorphism	R72 polymorphism	-
		c.521C>G, p.P174T	c.949_950insC	-
	**MYD88**	no mutations	no mutations	-
	**del17p13**	no	yes	-
**Cell markers**	**CD5**	95%	0%	0%
	**CD10**	0%	0%	0%
	**CD19**	100%	99%	99%
	**CD20**	92%	96%	96%
	**CD22**	98%	76%	67%
	**CD23**	90%	89%	36%
	**CD27**	98%	99%	-
	**CD38**	78%	90%	99%
	**CD45**	97%	99%	92%
	**CD80**	35%	98%	91%
	**CD83**	40%	4%	-
	**CD95**	90%	90%	63%
	**CD200**	26%	0%	-
	**IgD**	54%	97%	99%
	**IgM**	50%	99%	94%
	**HLA-DR**	43%	99%	97%
	**FMC7**	0%	8%	72%
	**ZAP70**	37%	18%	-
	**CXCR4**	9%	5%	-
	**CXCR5**	42%	12%	-
	**VLA4**	90%	98%	-
	**CD54**	72%	98%	98%
	**p-ERK**	87%	Positive WB ^28^	-
