# Supplementary figures and images for: Establishment and Characterization of PCL12, a Novel CD5+ Chronic Lymphocytic Leukaemia Cell Line
Source: PLoS One. 2015 Jun 25;10(6):e0130195. doi: 10.1371/journal.pone.0130195 (PMC4481539; doi:10.1371/journal.pone.0130195)

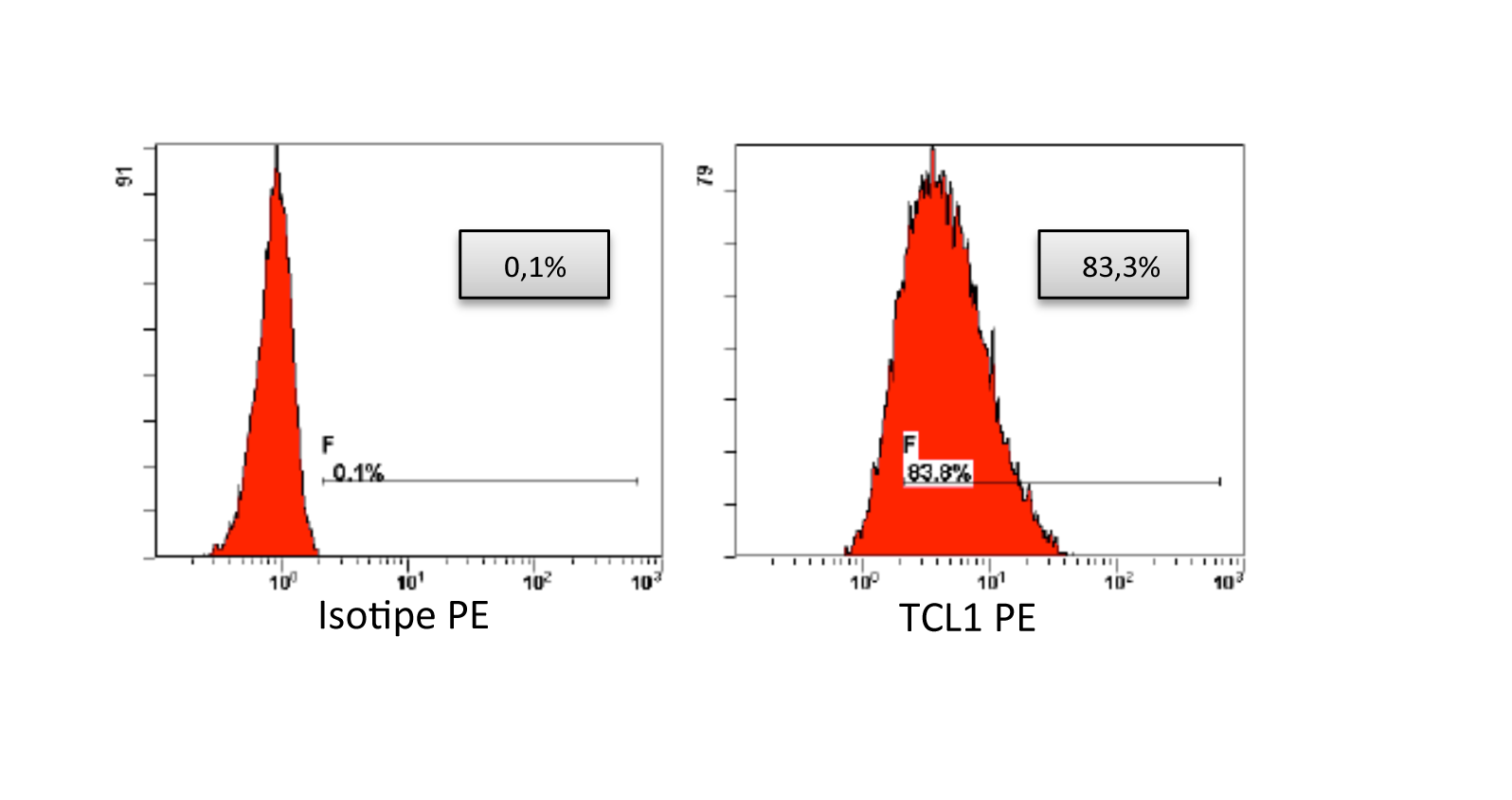

Supplement: S1 Fig — (TIF) [file pone.0130195.s001.tif]

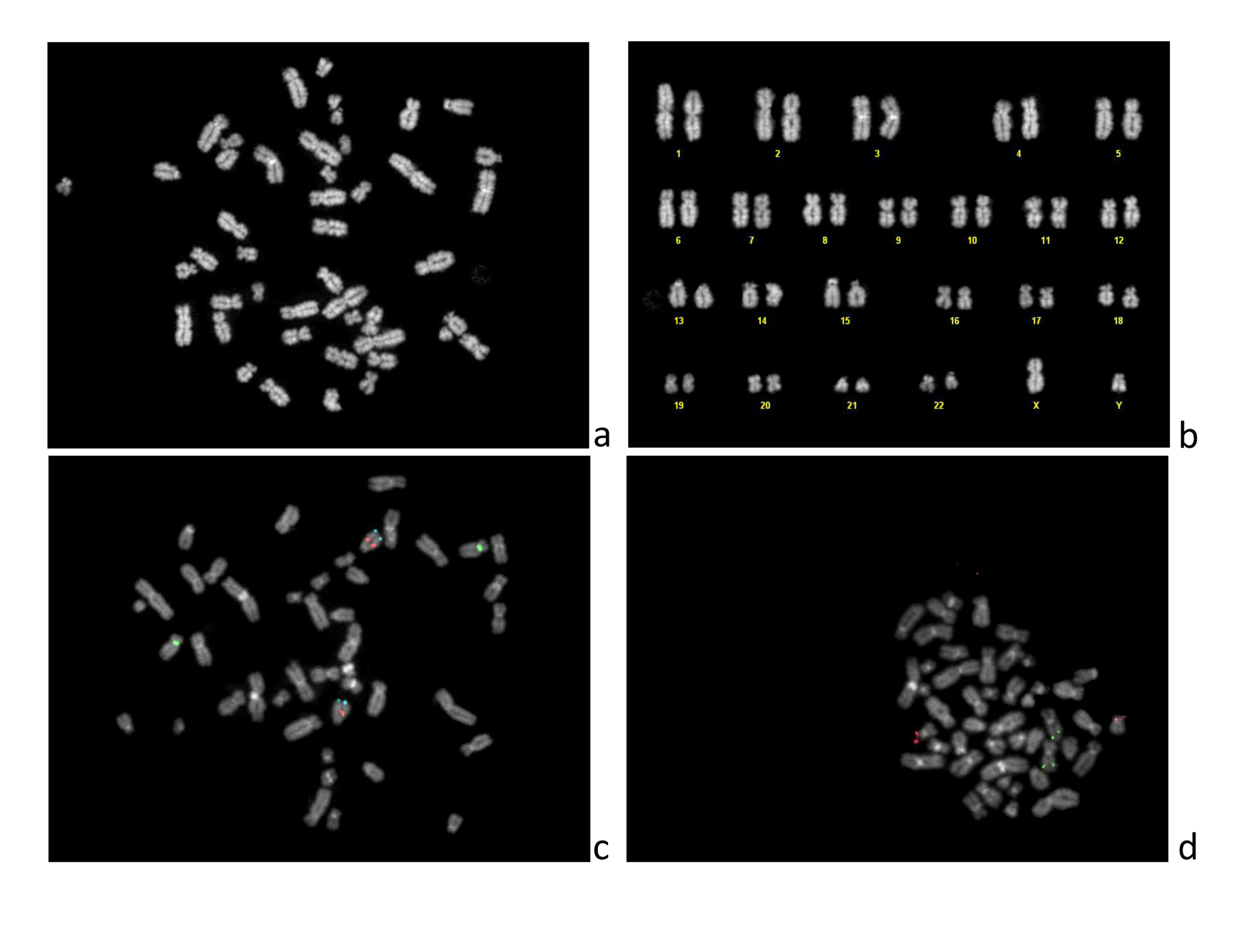

Supplement: S2 Fig — (TIF) [file pone.0130195.s002.tif]

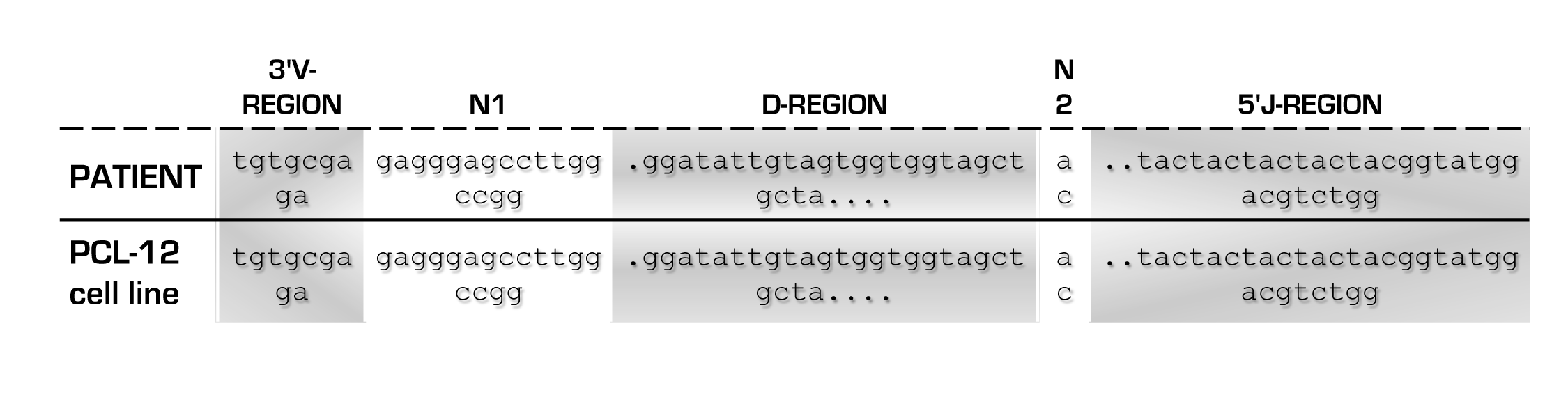

Supplement: S3 Fig — (TIF) [file pone.0130195.s003.tif]

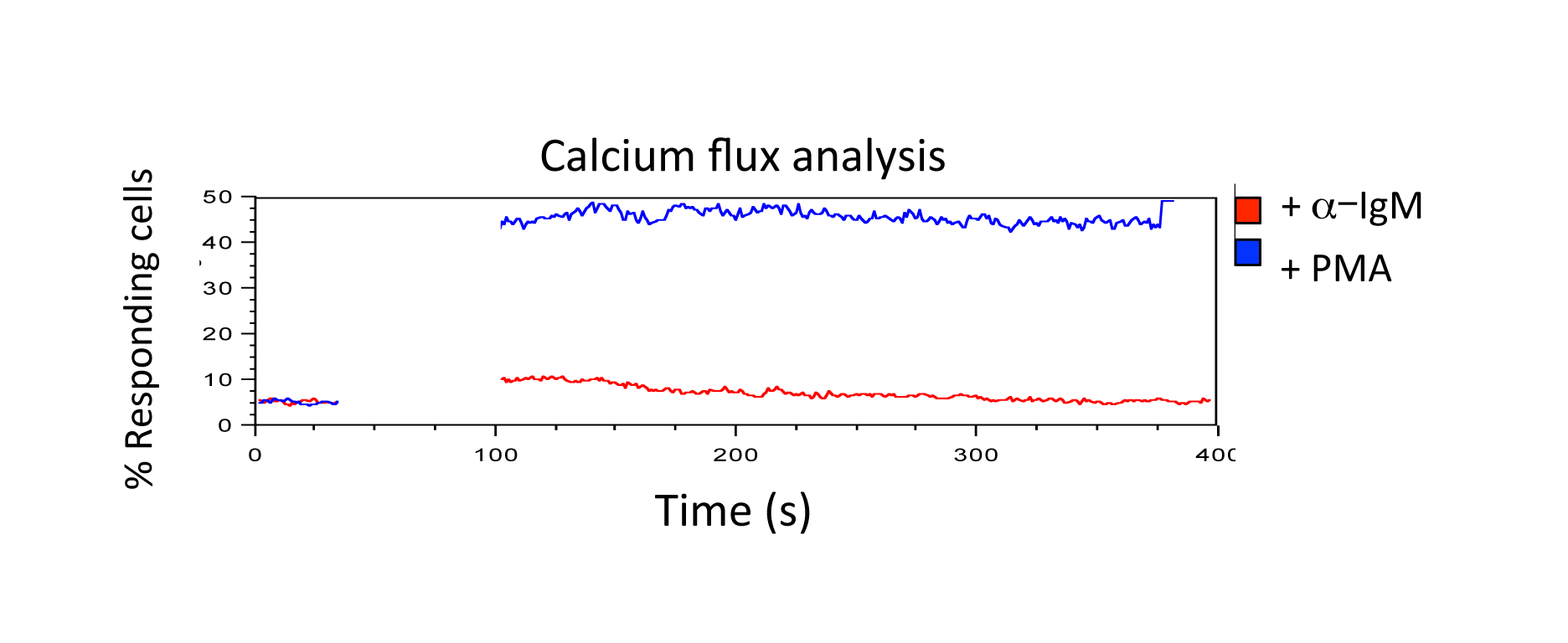

Supplement: S4 Fig — (TIF) [file pone.0130195.s004.tif]
